# Fecal Microbiota Transplantaion in the treatement of mood disorders : A literature review

**DOI:** 10.1192/j.eurpsy.2024.663

**Published:** 2024-08-27

**Authors:** F. Askri, K. Mahfoudh, K. Ben Younes, E. Herelli, U. Ouali, A. Aissa

**Affiliations:** ^1^PSYCHIATRY A; ^2^PSYCHIATRY C, RAZI HOSPITAL, MANOUBA, Tunisia

## Abstract

**Introduction:**

Many researchers have turned their attention to studying the relation between the gut microbiota to mood disorders. In fact, studies in the last 5 years have shown that the change in microbiota in animals can cause anxiety a depression –like behaviors.

In humans, considering the fact that there was a difference between in human gut microbiota between depressed persons and healthy controls, many clinicians suggest different treatment ways to compensate the microbiome imbalance such as Fecal microbiota transplantation (FMT).

FMT is an ancient tool that used to treat food poisoning and severe diarrhea. Recent studies have shown its efficacy in autism spectrum disorders but not enough studies have shown its contribution in treating mood disorders.

**Objectives:**

The aim is to explore and understand the use of fecal microbiota transplantaion in the mood disorder treatment

**Methods:**

We conducted a literature search for English articles on PubMed using the keywords : mood disorder, Fecal microbiota transplantation, treatment.

**Results:**

13 results were initially found on the pubmed database. we identified 4 eligible studies.

02 case studies reported that patients diagnosed with bipolar disorder type 2 improved after repetitive FMT treatment, 01 randomised controlled trial concluded good tolerability and feasibility of FMT in major depression disorder but was not designed to measure clinical outcomes. Finally, 01 study protocol is still conducting on the efficacy and safety of FMT n in a population with bipolar disorder during depressive episodes.

**Conclusions:**

No results have shown the efficacy of FMT in treating mood disorders yet. However, it is considered well tolerated and safe. Further studies are needed to conclude its efficacy.

**Disclosure of Interest:**

None Declared

